# Management of a Radicular Cyst in Anterior Maxilla With Endosurgical Intervention Along With Use of Mineral Trioxide Aggregate (MTA) and Bone Graft: A Case Report

**DOI:** 10.7759/cureus.47183

**Published:** 2023-10-17

**Authors:** Sambarta Das, Abhisek Das, Swagat Panda, Sradhashree Dipallini, Monika Mohanty, Priyankaa Das

**Affiliations:** 1 Conservative Dentistry and Endodontics, Hi-Tech Medical College and Hospital, Bhubaneswar, IND; 2 Conservative Dentistry and Endodontics, Hi-Tech Dental College and Hospital, Bhubaneswar, IND; 3 Conservative Dentistry and Endodontics, Kalinga Institute of Medical Sciences, Bhubaneswar, IND

**Keywords:** hydroxyapatite bone grafts, thermoplasticized gutta-percha, mineral trioxide aggregate, intracanal medicament, triple antibiotic, splinting, periapical lesion, apicoectomy

## Abstract

Radicular cysts are the most common cystic lesions that affect the jaws, which, though mostly asymptomatic, can be seen radiographically as an oval or pear-shaped unilocular radiolucency in the periapical region. Nonsurgical root canal procedures and periapical surgery followed by placement of bone substitute and bioceramic root-end filling material is generally the treatment of choice. This case report highlights the endosurgical management of long-standing trauma that led to a radicular cyst with respect to three maxillary anterior teeth in a young adult. The clinical and radiographic examination led to a provisional diagnosis of a radicular cyst, which was confirmed by biopsy. Non-surgical root canal treatment was performed with Mineral Trioxide Aggregate (MTA) as the apical barrier and surgical enucleation of the cyst was performed followed by placement of hydroxyapatite bone graft. Follow-ups till two years were done, which revealed the successful management of the case.

## Introduction

A common consequence of long-standing tooth decay or injury is the radicular cyst, which originates from Malassez's cell rests [1]. The primary occurrence site is typically the tips of the affected teeth [2]. According to Shear (1992), a higher prevalence has been noted in the maxillary front area, with a preference among males [1].

Small-sized periapical granulomas can often be managed conservatively through non-surgical endodontic procedures, which serve as the primary approach for addressing these conditions [3]. Oztan M (2002) has shown that larger periapical lesions, like cysts, respond well to non-surgical treatment with calcium hydroxide paste [4]. However, when root canal therapy is not feasible or proves ineffective, periapical surgery becomes a viable and dependable alternative [5]. Lee et al. (2014), in their retrospective observational study, found that the most common treatment method for radicular cysts involved removing the cyst through enucleation along with apicoectomy [6].

This case report presents a large radicular cyst involving three maxillary anterior teeth, i.e., teeth #21, #22, and #23 with tooth #22 having an open apex. Advanced radiological methods like Cone Beam Computed Tomography (CBCT) and histopathological investigation like biopsy, along with bioceramic restorative materials and bone grafts, contributed to successful endosurgical management of the case.

## Case presentation

A 19-year-old female patient presented at the Department of Conservative Dentistry and Endodontics with the primary concern of experiencing pain, swelling, and the discharge of pus in the upper left anterior area of her mouth for a duration of 4 months. She revealed a past incident of trauma that occurred 11 years ago.

**Figure 1:**
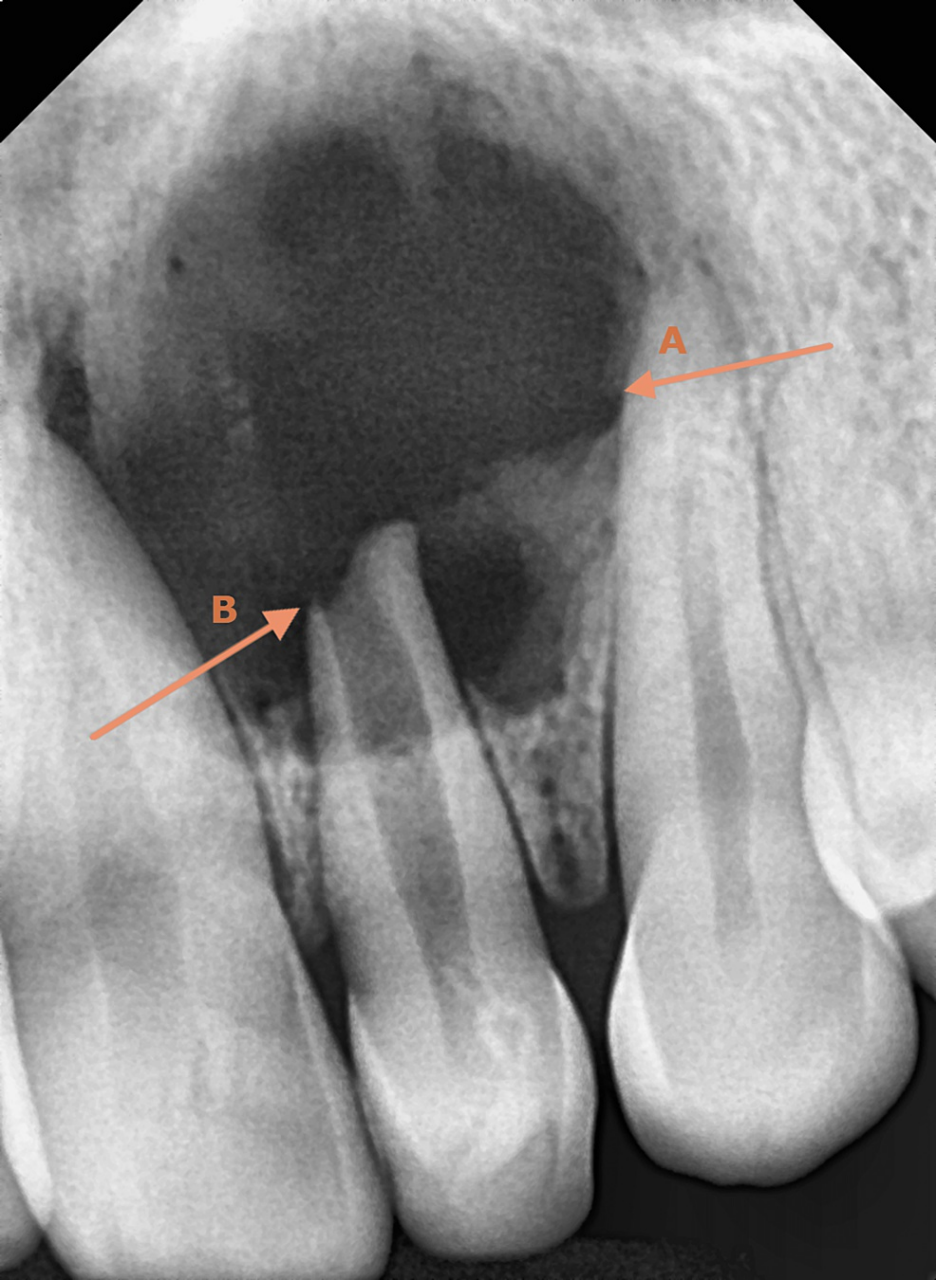
Digital X-ray before treatment A: Periapical radiograph showing a significant periapical radiolucency associated with teeth #21, #22, and #23 B: Tooth #22 with an open apex

Extraoral examination revealed evident facial asymmetry, with diffused swelling measuring 3x3 cm^2^ with obliterated nasolabial fold. On intraoral examination, a swelling on the labial vestibular region was appreciated. A heat vitality test was performed using a hot gutta-percha (GP) stick and electric pulp tester, which showed no response in these teeth. Radiological examination showed large periapical radiolucency in relation to teeth #21, #22, and #23 (Figure 1). Tooth #22 had an open apex which might be due to trauma at a very young age before root maturation.

The patient’s informed consent was obtained, and she was subjected to limited field of view (FOV) CBCT using Orthophos SL (Dentsply Sirona, USA) at a resolution of 80 µm to determine the extent of the lesion. The images revealed maxillary teeth #21, #22, and #23 having a single well-defined intra-bony periapical radiolucency measuring approximately 19-20 mm mesiodistally, 17.5mm supero-inferiorly, 13-14 mm bucco-palatally with distinct sclerotic border. Bucco-palatal extension of the lesion caused remarkable expansion and thinning of the palatal cortex followed by a breach in the palatal and buccal cortical plates. Patient history, and clinical and radiographic examinations led to a provisional diagnosis of infected radicular cyst.

The patient was explained about the condition, and the treatment plan was discussed. After obtaining informed consent, non-surgical endodontic treatment was initiated. An 18-gauge syringe was used to aspirate the cystic contents. The enucleated lining was transported in 10% formalin in an air-tight container for histological examination. Splinting was done using a fibre splint on the labial aspect from tooth #13 to #24 followed by access opening and biomechanical preparation. Due to continuous pus drainage through the root canal, the patient was to wait for some time. Once the pus drainage was reduced, a closed dressing was given with two small cotton balls in the access cavity sealed with triple antibiotic paste (temporary restoration). Oral antibiotics (amoxicillin 500mg along with clavulanic acid 125mg) were prescribed at a dosage of thrice daily for five days along with a combination of non-steroidal anti-inflammatory drugs (NSAID) (aceclofenac 100mg with serratiopeptidase 15mg and paracetamol 325mg) was given for twice daily for five days. The patient went through a complete oral prophylactic procedure before the next visit.

The patient was recalled after 48 hours to review the clinical condition. Temporary restoration was removed and the canals were irrigated using normal saline. Then the canals were dried using sterile paper points and a closed dressing with triple antibiotic paste was given. The patient was recalled after two weeks for obturation and periapical surgery.

During the subsequent appointment, the interim restoration was taken out, and the canals were flushed with chlorhexidine (CHX) 2% as a cleansing solution. Tooth #22 was carefully dried, and a 3mm apical barrier made of Mineral Trioxide Aggregate (MTA) was placed at the root tip using an MTA carrier. The filling of the root canal was accomplished using thermoplasticized gutta-percha and the patient was prepped for the surgical procedure. 

**Figure 2:**
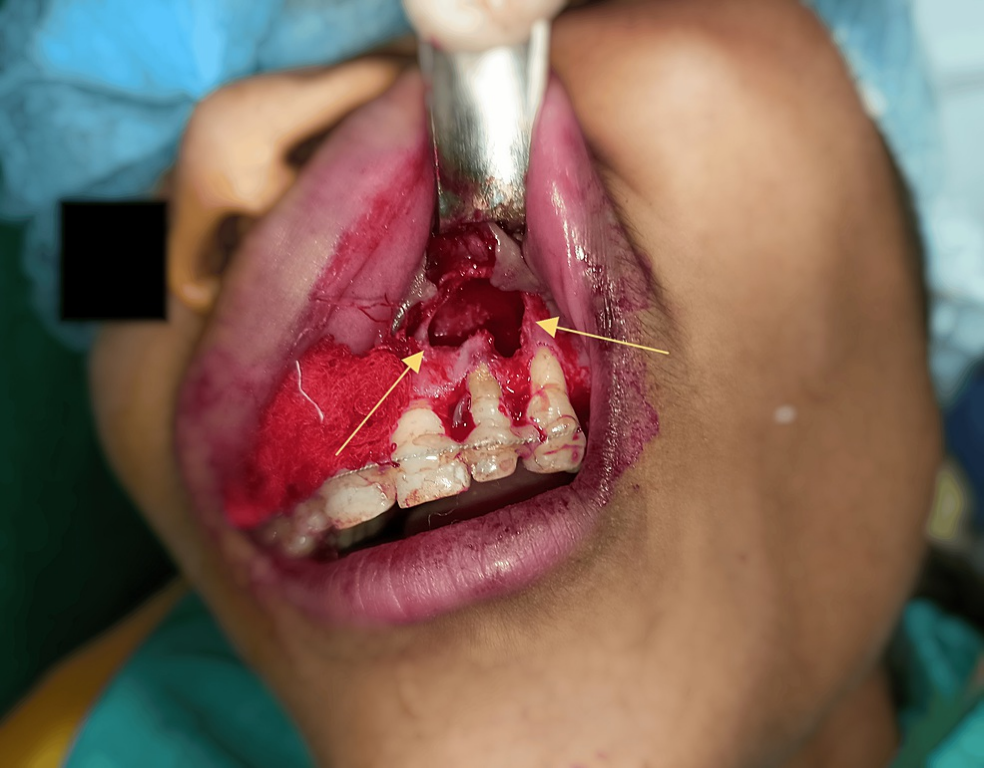
Flap reflection with cyst enucleation

For local anaesthesia, the infraorbital and nasopalatine nerve areas were numbed with a mixture of lignocaine and adrenaline at a ratio of 1:80,000. A complete mucoperiosteal flap was raised, extending from tooth #21 to #23. Upon elevating the flap, the cystic formation was clearly visible at the apex of tooth #22, along with a gap along the root's length. To excise the cyst, wet gauze was employed to create a separation between the cyst and the surrounding bone. With the utilization of our utmost surgical skill and expertise, the cyst was fully extracted and preserved in formalin for subsequent analyses (Figure 2).

**Figure 3:**
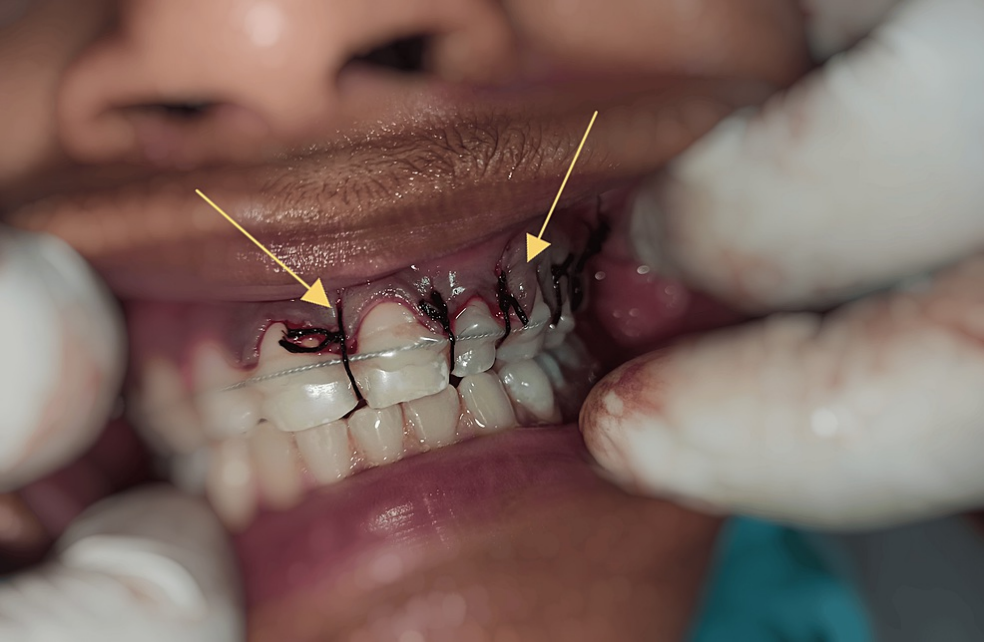
Flap reapproximation with suturing

To address the void left by the cyst's removal, a commercially available hydroxyapatite bone graft was inserted into the defect. The flap was then carefully repositioned, and sutures made of 3-0 black silk were used to close the incision (Figure 3).

The patient was instructed to use a 0.2% chlorhexidine gluconate mouthwash for the following five days to maintain oral hygiene. The removed lining of the cyst was submitted for histopathological analysis, which revealed a stratified squamous epithelium covering a connective tissue stroma. This underlying stroma had collagen fibres and a fibro cellular composition. Notably, there was a pronounced infiltration of chronic inflammatory cells and fibroblasts. This histopathological finding definitively supported our diagnosis of an infected radicular cyst.

**Figure 4:**
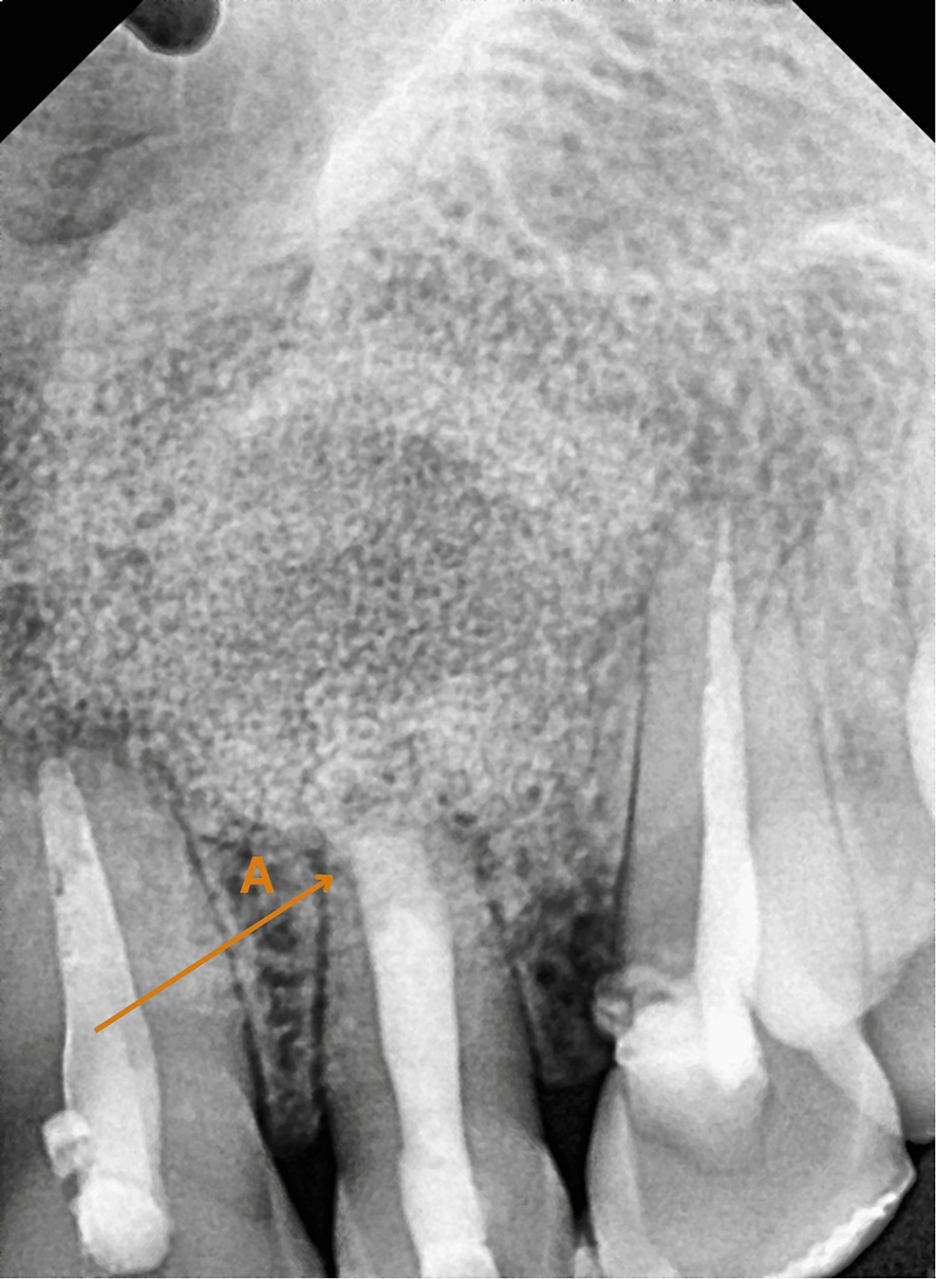
Digital X-ray after six months A: Mineral Trioxide Aggregate (MTA) apexification completed

**Figure 5:**
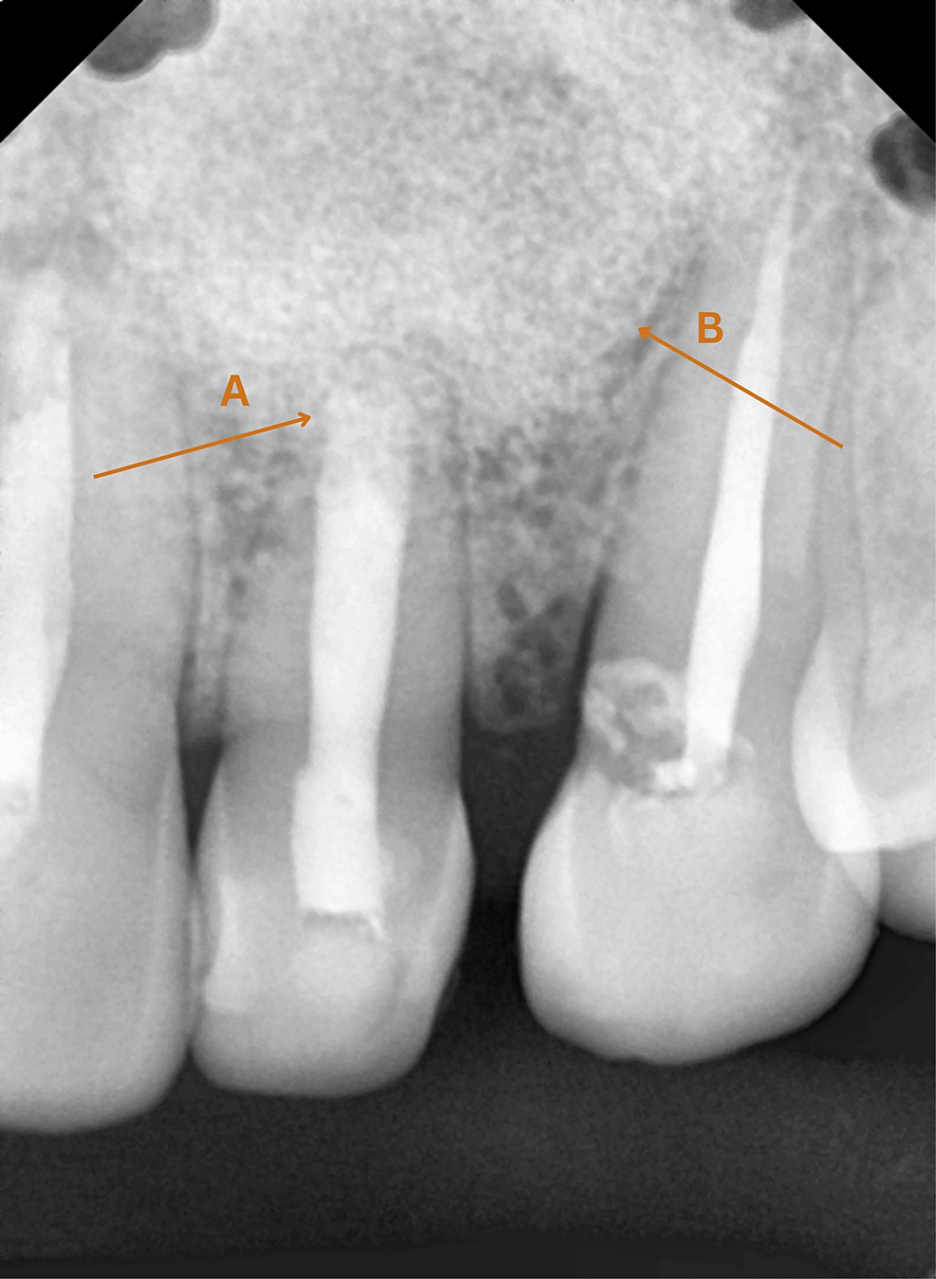
Digital X-ray after 18 months A: Mineral Trioxide Aggregate (MTA) apexification; B: Integration of bone graft

The patient was recalled at intervals of one, six, 12, and 18 months for follow-up reviews (Figures 4-5).

## Discussion

The radicular cyst is typically characterized as a fluid-filled cavity that arises from epithelial remnants (known as rests of Malassez) within the periodontal ligament [1]. It commonly develops as a result of inflammation, often following the necrosis of the dental pulp. While these cysts are frequently asymptomatic and identified through routine radiographic assessments, cases with prolonged existence might exhibit sudden aggravation of the cystic lesion, leading to symptoms like swelling, tooth mobility, and displacement of neighbouring unerupted teeth [7].

In such cases, surgical intervention followed by the application of suitable bone graft material is the preferred treatment approach [8]. A significant element in the success of surgical treatment lies in the choice of root-end filling material [9]. Combining root-end filling with gutta-percha obturation within the remaining root canal structure establishes an airtight barrier, preventing contact with infected periradicular tissues [10].

In the present case, MTA was employed as the retrograde filling substance. The selection of an ideal root canal filling material (thermoplastic GP) has a substantial impact on the surgical outcome. An optimal material should possess qualities such as non-absorbency, non-corrosiveness, non-cytotoxicity, resistance to moisture, dimensional stability, biocompatibility, antibacterial properties, radiopacity, cost-effectiveness, ease of manipulation, adhesion to dentinal walls, the establishment of a secure seal, and the initiation of cementogenesis [11,12].

In a study by Torabinejad et al. (1995), MTA demonstrated the prevention of apical leakage of introduced *Staphylococcus epidermidis* bacteria [13]. Moreover, MTA was found to encourage the formation of hard tissue and the regeneration of new cementum [14].

The process of bone regeneration following periapical surgery hinges on crucial factors such as primary wound closure, angiogenesis for blood supply, a source of undifferentiated mesenchymal cells, maintaining space, and ensuring wound stability (referred to as the PASS principle). The integration of bone grafts represents the most prevalent regeneration technique [15]. Osteoconductive hydroxyapatite, an alloplastic material, has been extensively used in periapical surgery to stimulate new bone formation [16]. It was therefore adopted in the current case to enhance bone regeneration.

## Conclusions

This case report illustrates the successful management of a complex radicular cyst involving multiple maxillary anterior teeth. It emphasizes the critical role of a multidisciplinary approach, advanced diagnostics, precise surgical and non-surgical interventions, and the strategic use of materials like MTA and hydroxyapatite bone graft. This case highlights the challenges in treating such cysts and stresses the importance of collaborative efforts among dental specialists. Long-term follow-up confirms treatment effectiveness, emphasizing the need for ongoing monitoring to assess healing and stability over time.
